# Multiplex-RT-PCR-ELISA panel for detecting mosquito-borne pathogens: *Plasmodium* sp. preserved and eluted from dried blood spots on sample cards

**DOI:** 10.1186/s12936-021-03595-4

**Published:** 2021-02-01

**Authors:** Philip Koliopoulos, Neema Mathias Kayange, Tim Daniel, Florian Huth, Britta Gröndahl, Grey Carolina Medina-Montaño, Leah Pretsch, Julia Klüber, Christian Schmidt, Antke Züchner, Sebastian Ulbert, Steven E. Mshana, Marylyn Addo, Stephan Gehring

**Affiliations:** 1 Center of Pediatric and Adolescent Medicine, University Medical Center, Mainz, Germany; 2 Department of Pediatric and Adolescent Medicine, Bugando Medical Centre, Mwanza, Tanzania; 3 Department of Microbiology and Immunology, Catholic University of Health and Allied Sciences, Mwanza, Tanzania; 4 Department of Pediatric and Adolescent Medicine, St. Vinzenz-Hospital, Dinslaken, Germany; 5 Fraunhofer Institute for Cell Therapy and Immunology, Leipzig, Germany; 6 Department of Infectiology and Tropical Medicine, University Medical Center Hamburg-Eppendorf, Eppendorf, Germany; 7 Department of Infection and Immunity, Luxembourg Institute of Health, Esch-sur-Alzette, Luxembourg

**Keywords:** Acute febrile diseases, *Plasmodium falciparum*, Sub-Saharan-Africa, Malaria rapid diagnostic test, Whatman filter cards, Dried blood spots, Mosquito-borne diseases, Dengue virus, Multiplex-RT-PCR-ELISA, Tanzania

## Abstract

**Background:**

Children are the most vulnerable group affected by malaria and other tropical, vector-borne diseases in low-resource countries. Infants presenting with acute onset fever represent a major sector of outpatient care in the Lake Victoria region. Misclassification and overuse of antibiotics and anti-malarial medications are consistent problems. Identifying the prevalent mosquito-borne pathogens in the region will reduce the prescription of non-indicated medicines.

**Methods:**

The literature was reviewed focusing on the mosquito-borne pathogens most prevalent in sub-Saharan Africa. Accordingly, an assay comprised of a multiplex-reverse transcriptase-polymerase chain reaction and an enzyme-linked immunosorbent assay (multiplex-RT-PCR-ELISA) was designed and validated in its ability to identify and differentiate nine human mosquito-borne pathogens including eight arboviruses and *Plasmodium* sp*.*, the aetiologic agents of malaria. Blood samples obtained from 132 children suspected of having malaria were spotted and preserved on Whatman^®^ 903 protein sample cards. Multiplex-RT-PCR-ELISA analysis was assessed and compared to results obtained by blood smear microscopy and the malaria rapid diagnostic test (RDT).

**Results:**

Nine out of nine pathogens were amplified specifically by the multiplex-RT-PCR-ELISA panel. Twenty-seven out of 132 paediatric patients presenting with acute fever were infected with *Plasmodium* sp*.*, confirmed by multiplex-RT-PCR. The results of blood smear microscopy were only 40% sensitive and 92.8% specific. The malaria RDT, on the other hand, detected acute *Plasmodium* infections with 96.3% sensitivity and 98.1% specificity. The preservation of *Plasmodium* sp*.* in clinical sera and whole blood samples spotted on sample cards was evaluated. The duration of successful, sample card storage was 186 to 312 days.

**Conclusions:**

Reliable, easy-to-use point of care diagnostic tests are a powerful alternative to laboratory-dependent gold standard tests. The multiplex-RT-PCR-ELISA amplified and identified nine vector-borne pathogens including *Plasmodium* sp*.* with great accuracy. Translation of improved diagnostic approaches, i.e., multiplex-RT-PCR-ELISA, into effective treatment options promises to reduce childhood mortality and non-indicated prescriptions.

## Background

Acute mosquito-transmitted febrile diseases are a serious threat to children in sub-Saharan Africa (SSA). According to the World Health Organization (WHO) World Malaria Report 2018, 266,000 children in Africa died from malaria making up for 61% off all malaria deaths worldwide [1].

Increased focus on *Plasmodium* sp*.*, the aetiologic agents of malaria, has led to a significant decline in the incidence of disease in Tanzania due mainly to prophylactic measures [2]. Four out of five cases of malaria identified by microscopic evaluation of blood smears in rural areas of Southern Tanzania are false positives [3]. “Negative syndrome” applies to children with malaria-like symptoms, but unconfirmed malaria. The malaria rapid diagnostic test (RDT) is a reliable, easy to use method that detects antigens specific to *Plasmodium* sp. Negative test results led to a decrease in the prescription of non-indicated, artemisinin-based anti-malarial drugs in low-resource settings [4].

Regrettably, an increasing number of malaria-like diseases caused by viral and bacterial pathogens have gained importance in recent years. Certain arboviruses, like Dengue (DENV) and Chikungunya (CHIKV) viruses, are responsible for huge epidemic outbreaks worldwide [5]. Since DENV is endemic in more than 125 countries, over 50% of the world’s population is at risk of infection [6]. The presence of DENV and CHIKV throughout sub-Saharan Africa (SSA) is likely and outbreak events are reported regularly, but reliable epidemiologic data are currently lacking [7, 8]. Inadequate treatment decisions are unavoidable due to the lack of diagnostic tools capable of distinguishing between pathogens [9]. Consequently, significant health, financial, and logistic burdens are inevitable for SSA communities [10]. Epidemiologic and diagnostic recognition of the relevant, circulating pathogens is indispensable for paediatric health care in the Lake Victoria Region, Tanzania. Moreover, it could inform the future aspects of arboviral spread to other continents [11–13].

A combination of a conventional multiplex-reverse transcriptase-polymerase chain reaction and an enzyme-linked immunosorbent assay (multiplex-RT-PCR-ELISA) in a microwell plate is a well-established, low-cost method used to detect multiple pathogens simultaneously [14]. The ability of multiplex-RT-PCR-ELISA to detect multiple pathogens in a single sample makes it highly valuable for distinguishing between different pathogens that cause acute febrile diseases. Unfortunately, current multiplex-RT-PCR methods focusing on arboviruses usually include DENV and CHIKV, but often omit other important pathogens, such as Zika virus (ZIKV), Rift Valley fever virus (RVFV) and Yellow fever virus (YFV) [15, 16].

Dried blood spots (DBS) on sample cards are a common approach used to preserve viral nucleic acids for subsequent diagnosis. DBS sample cards proved to be of tremendous value in epidemiologic studies that evaluated the prevalence of HIV, HCV, and HBV [17] and DENV [18], CHIKV [19] and *Plasmodium* sp. [20]. Notably, the WHO provided an algorithm in 2010 to detect treatment-resistant HIV strains by DBS-based RT-PCR [21]. Importantly, the WHO advises screening for “neglected tropical diseases” using developing technologies applicable to the most affected countries [22]. The preservation capacity of sample cards containing serum samples obtained from patients with arbovirus infections show concordance rates up to 95% [23].

The study described herein was undertaken to design a diagnostic test to identify, and distinguish between, the vector-borne pathogens capable of causing acute fever in children in SSA. A principal focus was optimizing the collection and storage of blood samples obtained from children in low resource settings like SSA. To this end, an established protocol for preparing and processing HIV-, HBV- and HCV-positive DBS samples was modified to accommodate the conditions found in SSA.

## Methods

### Patient population

A hospital-based cross-sectional prospective study was initiated at Bugando Medical Centre in Mwanza, Tanzania. Sociodemographic clinical data and blood samples were collected consecutively between April 2016 and October 2016 from 132 children, 5-months to 12-years of age. The children presented with acute high-grade fever (> 38.0 °C) and met the criteria of a presumptive malaria infection according to WHO definitions [24].

### Sample collection and storage

Venous blood (400 µl) was collected from each patient, centrifuged and stored at − 20 °C. The NADAL^®^ Malaria Pf/Pan Ag 4 Species Test (nal von minden GmbH, Regensburg, Germany) was performed and the results of blood smear microscopy for malaria were obtained at the respective health facility. Whatman^®^ 903 protein sample cards, spotted with patient whole blood or sera at the time of collection, were dried and prepared according to a modification of the protocol of Grüner described below [17]. Sample cards were stored at room temperature under dry conditions for 6- to 10-months in zip-locked plastic bags containing desiccant sachets. The frozen sera and spotted card samples were shipped to University of Mainz, Department of Pediatric Immunology and Infectious Diseases according to IATA and ICAO regulations.

### Sample card extraction

Whole blood and serum spotted on sample cards were processed by a modification of the methods reported in a study by Grüner et al*.*, which validated this approach in a comparison of 1,762 paired, serum and DBS samples [17]. DBS elution is the crucial step to generating intact nucleic acid and serologic markers for testing. DBS elution was optimized by spotting 50 µl whole blood obtained from *Plasmodium falciparum* and *Plasmodium vivax* patients on Whatman^®^ 903 protein sample cards. The cards were dried for 24 h and stored in zip-locked plastic bags for two weeks under dry condition at room temperature according to Grüner et al*.* [17]. The card punch procedure was standardized with a 3 mm wide hole. Six holes were punched from each sample blood spot, two from the centre and four from outer edge of the field; the six sample disks were transferred directly to a reaction tube. To prevent carryover and false positive results, card punches were rinsed sequentially for 30 s in the following solutions: distilled water; DNA-ExitusPlus™ (decontamination reagent, AppliChem GmbH, Darmstadt, Germany); distilled water and Terralin^®^ liquid (alcohol-based disinfectant, Schülke & Mayr GmbH, Norderstedt, Germany). The card punch was air-dried afterwards.

**Fig. 1:**
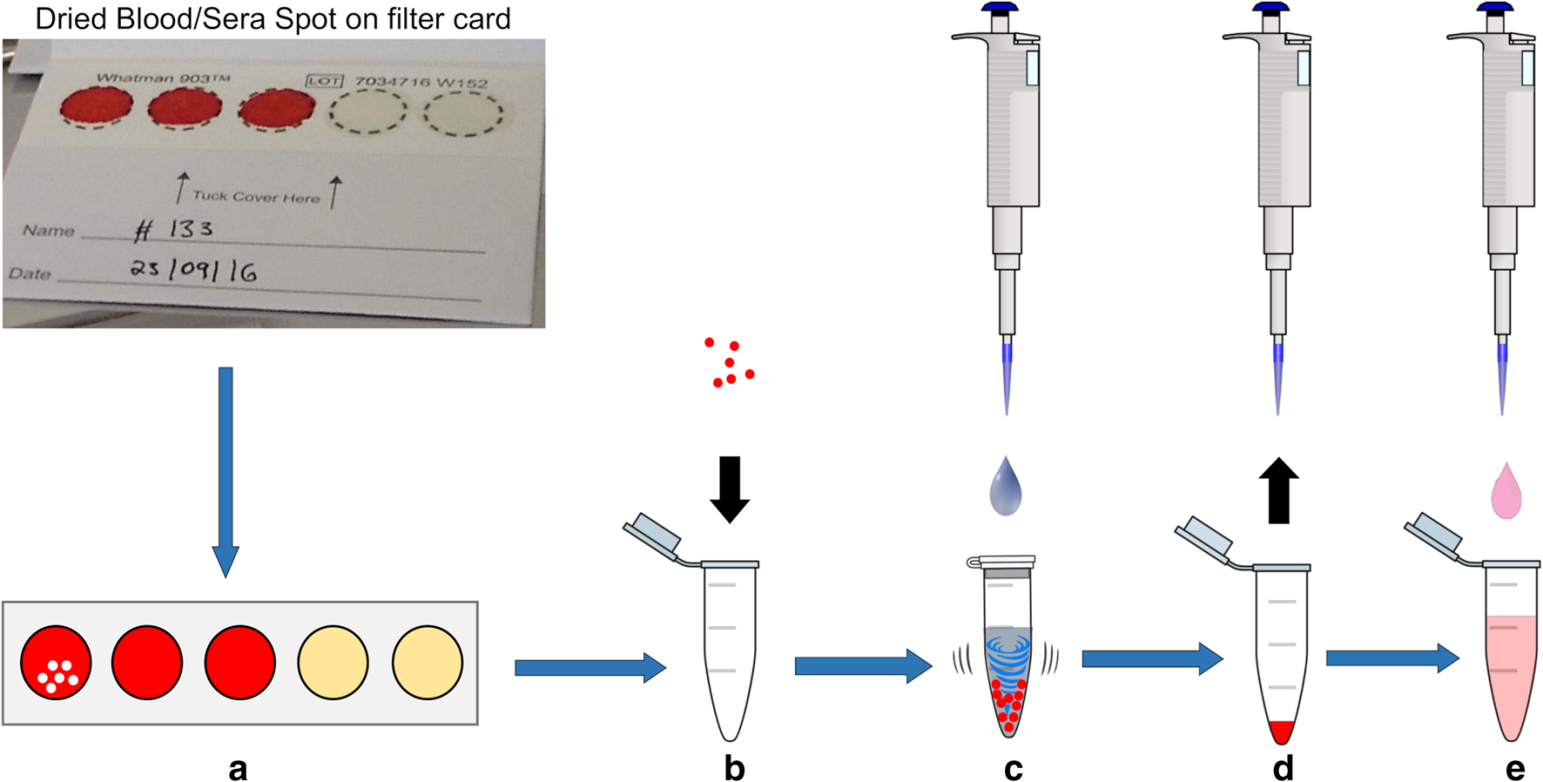
DBS elution from Whatman^®^ 903 protein sample cards. DBS were eluted from sample cards by the method of Klüber (Klüber, J.K., Master’s Thesis, unpublished): **a** make standardized holes with a punch; **b** transfer paper disks; **c** add nuclease free water and incubate for 60 min at 72 °C, vortex every 15 min; **d** collect the supernatant after centrifugation; **e** transfer supernatant to new tube and store at − 20 °C

The capacity of DBS to preserve pathogen-derived nucleic acid was validated by spotting positive controls on sample cards and testing three different elution methods (Fig. 1; Table 1). Incubation and shaking at high temperatures were performed in a heating block (Eppendorf^®^ Thermomixer^®^); overnight incubation at room temperature was performed in a three-dimensional shaker (TL10 Edmund Bühler). After elution, the sample was centrifuged for 3 min at 8000 rpm and the aqueous phase was transferred to a new reaction tube. The supernatant was stored at − 20 °C and multiplex-RT-PCR-ELISA was performed as outlined below within 2 months following extraction.

**Table 1:** Sample card extraction

	Klüber method^a^	Klüber-Grüner method	Grüner method [17]
Amount	Six paper disks (3 mm)	Six paper disks (3 mm)	Six paper disks (3 mm)
Time	1 h at 750 rpm	1 h at 750 rpm	Gently shaking overnight
Temperature	72 °C	72 °C	Room temperature
Buffer/process	300 μl of nuclease-free water. Vortex reaction tubes every 15 min	300 µl PBS-buffer containing Tween 20 and 0.9% NaCl. Vortex reaction tubes every 15 min	300 µl of PBS-buffer containing Tween 20 and 0.9% NaCl

Positive controls spotted on Whatman^®^ 903 protein sample cards were extracted by one of the three methods indicated

^a^J. Klüber, Master’s Thesis, unpublished

### Primers, probes and positive controls

Primer sets and biotinylated capture probes, selected based upon recent publications devoted to mosquito-transmitted pathogens in SSA (cited in Table 2), were purchased from ThermoFisher Invitrogen Custom DNA Oligonucleotides (Darmstadt, Germany). The influenza A primer set (primer 1, 5′-AAGGGCTTTCACCGAAGAGG-3′; primer 2, 5′-CCCATTCTCATTACTGCTTC-3′) and capture probe (5′-GTCAAGAGCACCGATTATCAC-3′), which were not components of the multiplex-RT-PCR-ELISA, were also purchased from ThermoFisher. The positive control for influenza A (inactivated virus particles) was obtained from Laboratory of Pediatric Immunology and Infectious Diseases, Mainz, Germany. Positive controls for all other organisms were obtained from the institutions listed Table 3. A singleplex-PCR validated the usefulness of the selected primers. The primers were subsequently incorporated into the multiplex-RT-PCR-ELISA panel for malaria-like diseases. To cover a broad range of mosquito transmittable diseases without overloading the multiplex-RT-PCR master mix, primer pairs covering DENV 1–4 and the clinical relevant *Plasmodium* sp*.* were included into the multiplex-RT-PCR-panel [25, 26] (Table 3). Remarkably, the Plasmo Plu3 primerset targets genus-specific the plasmodium 18S rRNA Gene and thereby grants enhanced sensitivity through the RT-step.

**Table 2:** Multiplex-RT-PCR-ELISA: primers and probes

	Family	No	Pathogens and prevalence	Primer	5′-3′ sequence	Concentration ( pm/µl)	Literature^a^
Arboviruses	Flaviviridae	1	Dengue virus (DENV) [8, 42, 61, 62]	C-prm D1	TCAATATGCTGAAACGCGAGAGAAACCG	5.5	[25]^b,c^
				D2	TTGCACCAACAGTCAATGTCTTCAGGTTC	5.5	
			DENV-1^d^	rTS1 (DEN-1)	CCCGTAACACTTTGATCGCT	5.5	
			DENV-2^d^	mTS2 (DEN-2)	CGCCACAAGGGCCATGAACAGTTT	5.5	
			DENV-3^d^	TS3 (DEN-3)	TAACATCATCATGAGACAGAGC	5.5	
			DENV-4^d^	rTS4 (DEN-4)	TTCTCCCGTTCAGGATGTTC	5.5	
		2	West Nile virus (WNV) [63–65]	WNV lancio F	CAGACCACGCTACGGCG	5.5	[66]^b^
				WNV lancio R	CTAGGGCCGCGTGGG	5.5	
				WNV lancio P	TCTGCGGAGAGTGCAGTCTGCGAT	10	
		3	Zika virus (ZIKV) [67, 68]	ZVforw	CAGCTGGCATCATGAAGAAYC	5.5	[70]^b^
				ZVrev1	CACTTGTCCCATCTTCTTCTCC	5.5	
				ZVrev2	CACCTGTCCCATCTTTTTCTCC	5.5	
				ZVprobe	CYGTTGTGGATGGAATAGTGG	10	
		4	Yellow Fever virus (YFV) [69–71]	YFV-F	GCACGGATGTAACAGACTGAAGA	5.5	[72]^b^
				YFV-R	CCAGGCCGAACCTGTCAT	5.5	
				YFV-probe	CGACTGTGTGGTCCGGCCCTC	10	
	Togaviridae (Alphaviruses) [37]	5	Semliki Forest virus (SFV) [64]	SFV-F	ACAGACTGTCACTGAGCAG	5.5	[75]^b^
				SFV-R	AGCTCCACGTCATCATTGAG	5.5	
				SFV-probe	GTGACCATCTACTGCAGAGA	10	
		6	O´nyong-nyong virus (ONNV) [74–76]	ONNV-F	GCAGGGAGGCCAGGACAGT	5.5	[79]^b^
				ONNV-R	GCCCCTTTTTCYTTGAGCCAGTA	5.5	
				ONNV-probe	TGTATTGCTCCTGCCGCTGG	10	
		7	Chikungunya virus (CHIKV) [42, 76, 78, 79]	ChikS	TGATCCCGACTCAACCATCCT	5.5	[82]^b^
				ChikAs	GGCAAACGCAGTGGTACTTCCT	5.5	
				ChikP	TCCGACATCATCCTCCTTGCTGGC	10	
	Bunyaviridae	8	Rift Valley fever virus (RVFV) [81, 82]	RVF-F	TGAAAATTCCTGAGACACATGG	5.5	[79]^b^
				RVF-R	ACTTCCTTGCATCATCTGATG	5.5	
				RVF-probe	CACAAGTCCACACAGGCCCCTTACAT	10	
Protozoa	Malaria (*Plasmodium* sp.)	9	*Plasmodium* sp. (MAL) [83]	Plasmo Plu3 F	GCTCTTTCTTGATTTCTTGGATG	5.5	[26]^b^
				Plasmo Plu3 R	AGCAGGTTAAGATCTCGTTCG	5.5	
				Plasmo Plu3 P	ATGGCCGTTTTTAGTTCGTG	10	
			*P. falciparum* [84]	FAL-F	CTTTTGAGAGGTTTTGTTACTTTGAGTAA	5.5	[51]^e^
				FAL-R	TATTCCATGCTGTAGTATTCAAACACAA	5.5	
				FAL-probe	TGTTCATAACAGACGGGTAGTCATGATTGAGTTCA	10	
			*P. vivax* [85]	VIV-F	ACGCTTCTAGCTTAATCCACATAACT	5.5	[88]^e^
				VIV-R	ATTTACTCAAAGTAACAAGGACTTCCAAGC	5.5	
				VIVAX-probe	TTCGTATCGACTTTGTGCGCATTTTGC	10	
			*P. malariae *[87, 88]	Pm-1	AGTTAAGGGAGTGAAGACGATCAGA	5.5	[89]^e^
				Pm-2	CAACCCAAAGACTTTGATTTCTCATAA	5.5	
				Pm-probe	ATGAGTGTTTCTTTTAGATAGC	10	

^a^The primers and probes listed were obtained from the literature cited

^b^Included in the multiplex-RT-PCR-ELISA

^c^Used to type DENV1-4 according to the method of Chien et al*.* [25]

^d^Probes for DENV1-4 consisted of the respective biotinylated primers

^e^Used to differentiate *Plasmodium* sp*.* by multiplex-RT-PCR-ELISA

**Table 3:** Multiplex-RT-PCR-ELISA positive controls

	Family	No	Pathogen	Source	Positive control
Arbo viruses	Flaviviridae	1	Dengue virus (DENV)	Laboratory of Pediatric Immunology and Infectious Diseases, Mainz, Germany	Inactivated particles: DENV serotypes 1, 2, 3 and 4
		2	West Nile virus (WNV)	HiSS Diagnostics GmbH, Freiburg im Breisgau, Germany	Inactivated cell culture supernatant: Accu Type TM West Nile virus lineage I
		3	Zika virus (ZIKV)	Robert Koch Institute, Berlin, Germany	Inactivated cell culture supernatant (strain MR766-infected Vero E6 cells); aliquoted and freeze-dried
	Togaviridae (Alphaviruses)	4	Yellow Fever virus (YFV)	Robert Koch Institute, Berlin, Germany	Inactivated cell culture supernatant
		5	Semliki Forest virus (SFV)	European Virus Archive goes Global, Marseille, France	Original isolate. RNA prepared from cell culture supernatant using QIAamp Viral RNA Mini Kit, Qiagen
		6	O´nyong-nyong virus (ONNV)	European Virus Archive goes Global	Inactivated cell culture supernatant (strain Dakar 234-infected Vero cells); freeze-dried sample
		7	Chikungunya virus (CHIKV)	Institut for Virology- Universitätsklinikum, Freiburg, Germany	Inactivated virus particles
	Bunyaviridae	8	Rift Valley Fever virus (RVFV)	Robert Koch Institute, Berlin, Germany	Inactivated cell culture supernatant
Protozoa	Malaria (*Plasmodium* sp.)	9	*P. falciparum* *P. vivax* *P. malariae*	Discovery Life Sciences, Los Osos, CA, USA	Serum obtained from patients with acute malaria infection (*P. falciparum, P. vivax,* and *P. malariae*). Microscopy results: parasite-positive

*Plasmodium* sp. (*P. falciparum, Plasmodium ovale, Plasmodium malariae*) were differentiated in a trivalent-RT-PCR. Positive controls, whole blood obtained from patients infected with *Plasmodium* sp. listed (Discovery Life Science, Inc., Los Osos, TX, USA), were tested. DENV serotyping using inactivated DENV 1–4 was performed subsequent to multiplex-RT-PCR to delineate the epidemiology of DENV infections in SSA.

### Multiplex-RT-PCR-ELISA

Total nucleic acid was isolated with the High Pure Viral Nucleic Acid Kit according to the manufacturer's instructions (Roche Diagnostics, Mannheim, Germany). Purified nucleic acid (4.5 µl) was used as the template for multiplex-RT-PCR-ELISA in accordance with methods described previously [14, 27, 28]. Target sequences for PCR were selected from the literature (Table 2).

The PCR product was denatured with NaOH and quantified by adding 15 μl/well to streptavidin-coated microtitre plates. The 3′biotinylated capture probe specific for the amplified target sequence (also listed in Table 2) was then added and the plates were incubated for at least 1.5 h at 37 °C. After washing, anti-digoxigenin-peroxidase (Roche Diagnostics) was added and the plates were reincubated for 45 min and washed. ABTS substrate solution (Roche Diagnostics) was added to each well, and the plates were incubated for another 30 min at 37 °C. The optical density (OD_405_), 492 nm reference filter, was determined. Positive and negative controls were included in each assay. The results were considered valid if all negative control values were ≤ 0.2 OD_405_. Samples were classified as PCR-positive or -negative dependent upon a 0.4 OD_405_ cut-off value; samples with initial values of 0.2–0.4 OD_405_ were considered borderline and only classified as positive or negative after retesting with a singleplex PCR approach.

The sensitivity of the multiplex-RT-PCR-ELISA panel was determined using ZIKV strain MR766 (1 × 10^7^ viral RNA copies/ml) obtained from the Robert Koch Institute as a positive control. Viral RNA was mixed with nuclease free H_2_O and serially diluted 10-fold to establish the minimal detectable viral copies. Multiplex-RT-PCR-ELISA was performed on serial dilutions of the ZIKV standard in a master mix containing the 9 primer pairs described above. Specificity was evaluated by quantifying the OD-values of the nine-fold hybridized PCR product.

Multiplex-RT-PCR-ELISA was performed on all frozen sera and samples derived from DBS and DSS. All samples, which were positive for mosquito-borne disease, were retested by multiplex-RT-PCR-ELISA and verified by a singleplex PCR approach (Plasmo Plu3 primerset, Table 2) using the primer set found to be the most sensitive among seven published sets targeting *Plasmodium* sp*.* or *P. falciparum* [29]. The patients’ clinical information, RDT and blood smear test results were known to the assessor of Multiplex-RT-PCR-ELISA performed on patients’ filtercards and sera samples. Contrariwise Multiplex-RT-PCR-ELISA test results of patients’ samples were not known to the performer of mRDT or blood smear tests.

### Biosafety

The isolation and extraction of pathogen nucleic acid were conducted following strict biosecurity standards in a biosafety level 2 cabinet. Each series of 9 specimens included one negative control (NaCl) to monitor cross-contamination. Nucleic acid extractions and the preparation of PCR-reagents were carried out in different rooms. Terralin^®^ liquid and UV light (30 min) were used at the end of each step to negate contamination [30].

### Statistical analysis

The sensitivity and specificity of two standard diagnostic tests for malaria (i.e., blood smear microscopy and the RDT) were evaluated using two-by-two tables comparing the results of each test with those obtained by multiplex-RT-PCR-ELISA on serum samples derived from the same patient. An interrater reliability analysis was conducted using the Cohen’s kappa statistic to determine consistency among the preservation methods, i.e., DBS versus DSS. The results were classified according to Byrd et al*.*: κ = 0.93–1.0, excellent; κ = 0.81–0.92, very good; κ = 0.61–0.80, good; κ = 0.41–0.60, satisfactory; κ = 0.21–0.40, slight; κ = 0.01–0.20, weak; and κ ≤ 0, no agreement [31]. The data were analysed with two statistical programs: SPSS 25 (IBM SPS Statistics, Armonk, New York, USA) and Sigma Plot 11 (Systat Software GmbH, Erkrath, Germany).

## Results

**Fig. 2:**
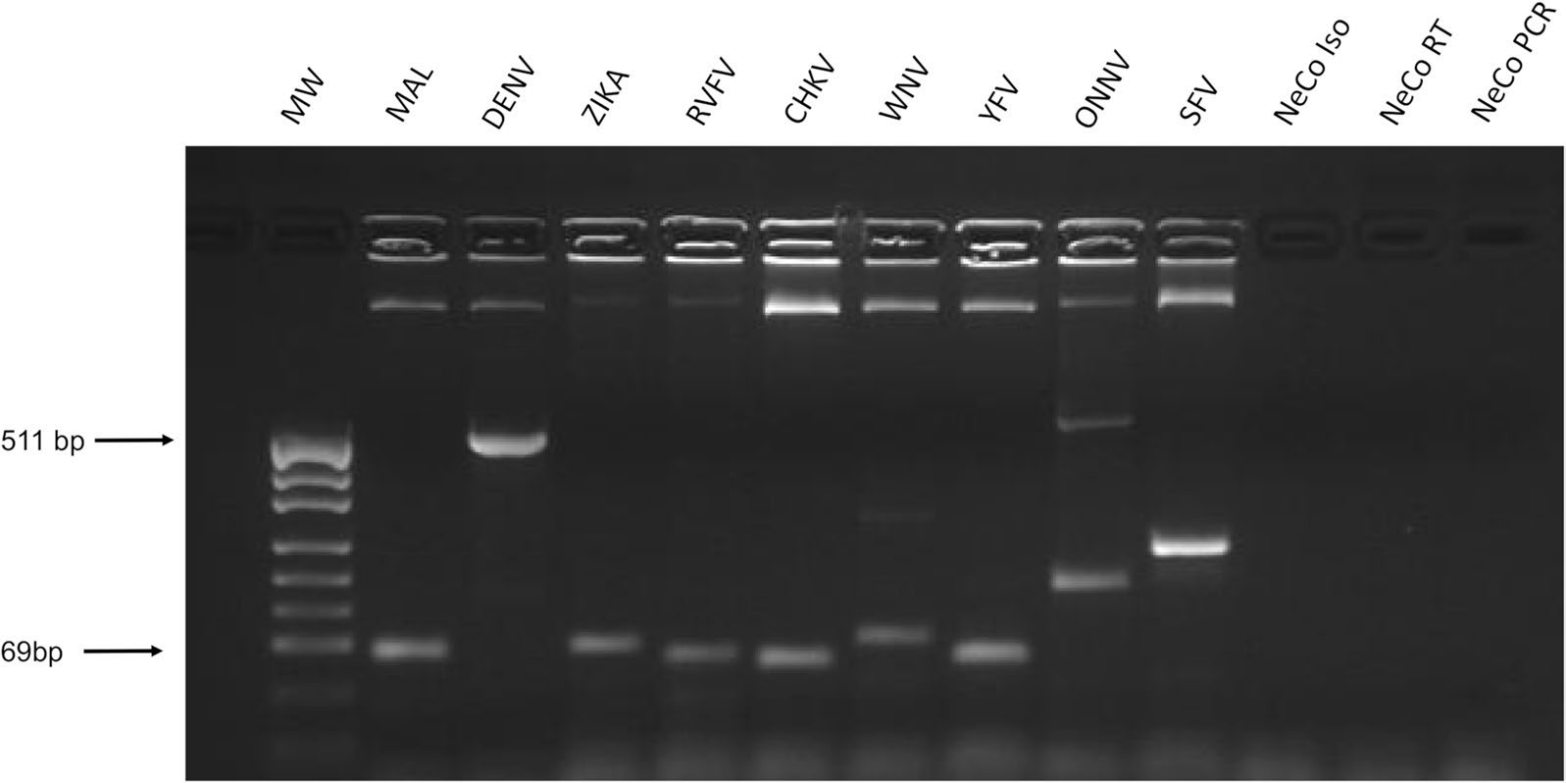
Gel electrophoresis of multiplex-RT-PCR products. The nucleic acid isolated from each of nine vector-borne pathogens (positive controls) was amplified using the multiplex-RT-PCR, and the product of each reaction was subjected to 2% agarose gel electrophoresis. *MW* molecular weight marker pUC19 DNA/*Msp*I, ThermoFisher Scientific, Waltham, MA USA) and the following pathogens, *MAL* malaria, 99 bp, *DENV* Dengue virus, 511 bp, *ZIKA* Zika virus, 100 bp, *RVFV* Rift Valley Fever virus, 69 bp, *CHIKV* Chikungunya virus, 81 bp, *WNV* West Nile virus, 116 bp, *YFV* Yellow Fever virus (YFV 83 bp), *ONNV* O´nyong-nyong virus, 148 bp, *SFV* Semliki Forest virus, 230 bp, *NeCo Iso* negative isolation control, *NeCo RT* negative reverse transcriptase control, *NeCo PCR* negative primermix control

**Fig. 3:**
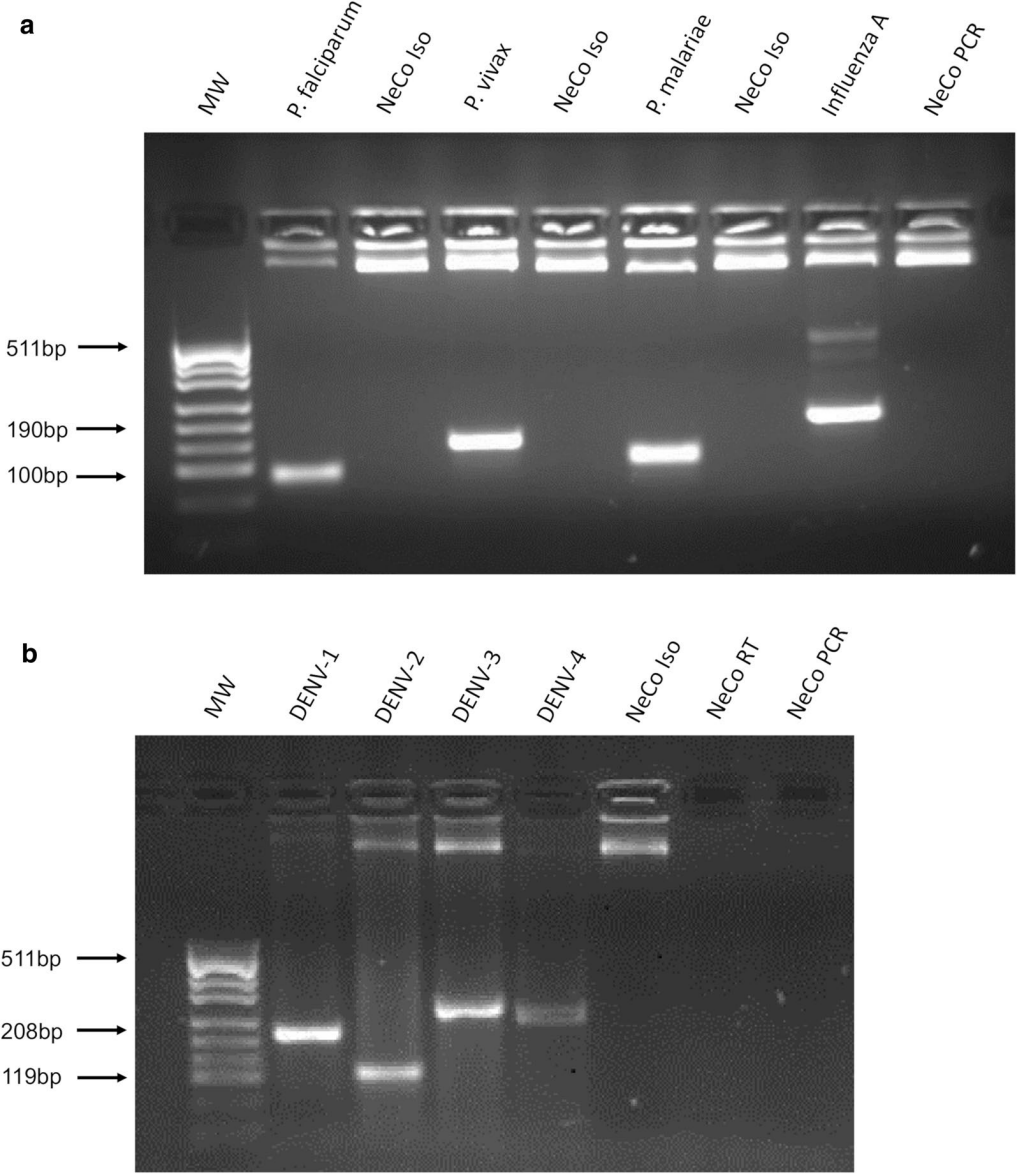
Differentiation of *Plasmodium* sp. and DENV serotypes. *Plasmodium falciparum, P. vivax* and *P. malariae* in species-positive sera derived from infected patients were amplified by multiplex-RT-PCR and then differentiated by 2% agarose gel electrophoresis (**a**). Inactivated DENV particles were amplified and serotyped by singleplex PCR (**b**). *MW* molecular weight marker pUC19 DNA/*Msp*I and the following species or serotypes: *P. falciparum*, 100 bp; *P. vivax*, 141 bp; *P. malariae*, 166 bp; DENV-1, 208 bp; DENV-2, 119 bp; DENV-3, 288 bp; and DENV-4, 260 bp; Influenza A, 190 bp served as a positive control; NaCl and nuclease-free H_2_O served as negative isolation (NeCo Iso) and primer mix controls (NeCo PCR), respectively

The ability to detect the following mosquito-borne pathogens prevalent in SSA was determined: *Plasmodium* sp*.*, DENV, WNV, ZIKV, YFV, SFV, ONNV, CHIKV and RVFV. The selected primer pairs listed in Table 2 were first tested in a singleplex PCR and then used in a nine-plex-RT-PCR, to detect a positive control. Gel electrophoresis documented that each targeted pathogen could be detected and amplified; the sizes of the PCR products of a single multiplex-RT-PCR run are shown in Fig. 2. The occurrence of unspecific bands underline that pathogens could not be differentiated based solely upon the size of the PCR product, necessitating analysis of the PCR products by hybridization with targeted, 3′biotinylated capture probes. The probes hybridized only with the multiplex-RT-PCR product (amplicon) of the positive control for which they were specific and meant to target. Hybridization with the amplicons generated from negative and unrelated positive controls was negligible. The resulting, normalized OD values are shown in Table 4. All OD values measured for the amplicons of the respective positive control were highly significant (≥ 0.4 OD). These findings evidence that each sequence was amplified specifically, and no cross-contamination occurred. *Plasmodium* sp. (*P. falciparum, P. vivax* and *P. malariae*) were differentiated using a multiplex-RT-PCR that recognized the three species in positive sera derived from different patients with acute malaria infections (Fig. 3). DENV-serotyping on inactivated viral particles was performed by singleplex PCR following Chien et al*.* [25].

**Table 4:** Multiplex-RT-PCR-ELISA

Positive control	*P. fal*	DENV^a^	ZIKA	RVFV	CHIKV	WNV	YFV	ONNV	SFV
ELISA^b^ (OD_405_)	2.31 ± 0.01	1.65 ± 0.07	2.12 ± 0.05	1.72 ± 0.09	1.27 ± 0.04	1.67 ± 0.11	2.04 ± 0.08	1.77 ± 0.07	1.92 ± 0.20

^a^DENV-2 serotype

^b^Data, normalized based upon the OD_405_ values generated from pooled influenza A amplicons, are the means ± SEM obtained from triplicate plates. OD readings ≥ 4-times the negative control (0.1 OD_405_) were judged to be positive [27]

### Evaluation of sensitivity

The ability of the multiplex-RT-PCR-ELISA panel to detect ZIKV RNA serially diluted from 1:10^6^ to 1:10^1^ copies/ml was determined in order to establish the limits of multiplex-RT-PCR sensitivity (Table 5). One thousand copies/ml was the limit of ZIKV RNA detection. OD-values considered borderline were only classified as positive or negative after retesting. Therefore, the sensitivity limit of the nine-valent, multiplex-RT-PCR-ELISA can be assumed to be up to 1000 copies of pathogen nucleic acid per ml. Detection limits were only determined for ZIKV; the concentration of viral RNA in the positive controls for the other arboviruses was not known. All OD-values were quantified in a single microwell-plate negating any need to normalize.

**Table 5:** Multiplex-RT-PCR-ELISA detection limit Zika-Virus^a^

Virus RNA copies/ml	1 × 10^6^	1 × 10^5^	1 × 10^4^	1 × 10^3^	1 × 10^2^	1 × 10^1^	1 × 10^0^
ELISA^b^ (OD_405_)	1.45 ± 0.05	1.55 ± 0.19	1.06 ± 0.17	0.41 ± 0.11	0.17 ± 0.13	0.09 ± 0.01	0.09 ± 0.03

^a^The MR766 strain of ZIKV (1 × 10^7^ viral RNA copies/ml) was serially diluted 1:10 and multiplex-RT-PCR-ELISA was conducted to determine the limits of detection

^b^Values are the means ± SEM calculated from 3 determinations. OD readings ≥ 4-times the negative control (0.1 OD_405_) were judged positive [27]. Values 2- to 4-times the negative control were considered borderline

### Extraction of positive controls from sample cards

**Fig. 4:**
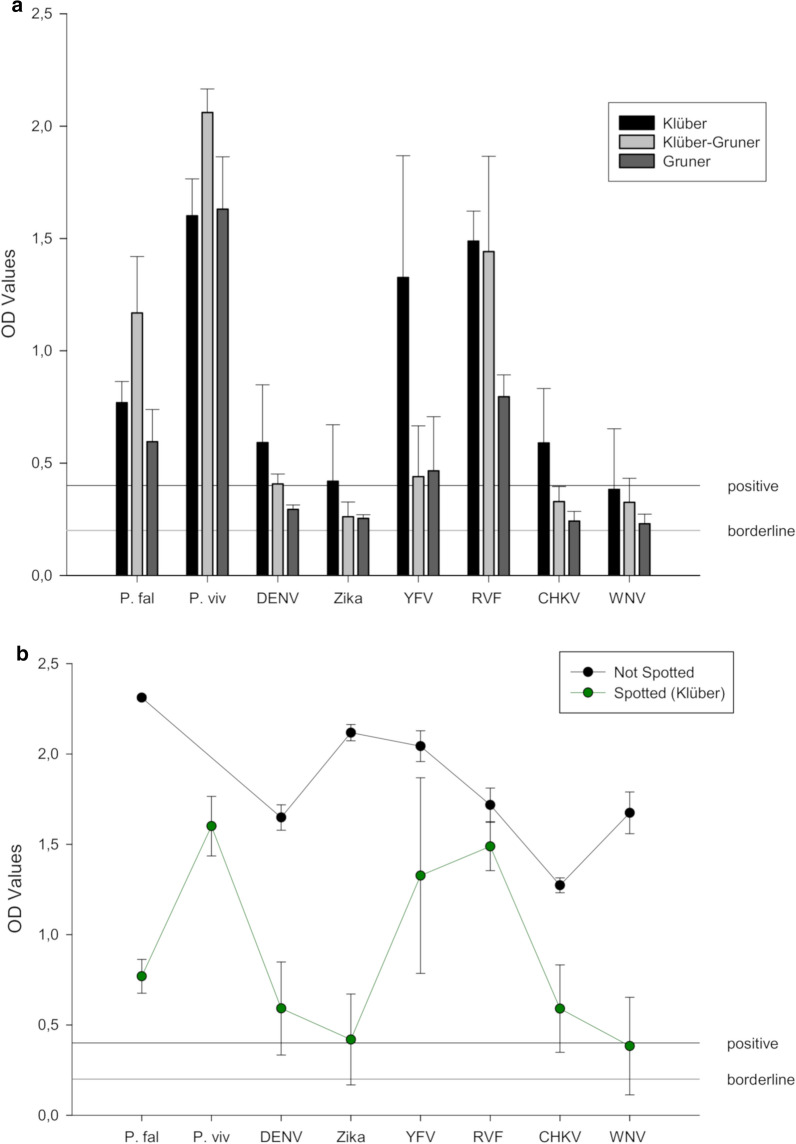
Efficacy of sample card extraction. Sample cards spotted with the positive controls listed on the x-axis were extracted by one of three methods indicated in the Key (**a**). Positive controls were either not spotted or spotted on sample cards and eluted by the method of Klüber (unpublished) (**b**). In all cases, the positive control in the resultant sample was quantified by multiplex-RT-PCR-ELISA. OD-values are the means ± standard deviation of triplicate determinations

The six paper disks derived from positive controls spotted on sample cards were eluted by 3 different elution methods described above. After incubation, the eluate was transferred into a new reaction tube, the nucleic acid was extracted, and multiplex-RT-PCR-ELISA was performed. According to the optical density values derived, six out of eight vector-borne pathogens extracted by the method of Klüber (Master’s Thesis, unpublished) using nuclease-free water were reliably detected (Tables 6, 7). The method of Grüner and co-workers [17] and a combination of the Klüber and Grüner methods could only extract four out of eight pathogens from sample cards (Table 6; Fig. 4a, b).

**Table 6:** Efficacy of multiplex-RT-PCR-ELISA conducted on positive controls eluted from sample cards

Pathogens^a^	*P. fal*	DENV	ZIKA	RVF	CHIKV	WNV	YFV
Spotted ELISA (OD_405_)	0.77	0.59	0.42	1.49	0.59	0.38	1.32
Not spotted ELISA (OD_405_)	2.31	1.65	2.12	1.72	1.27	1.67	2.04

^a^The positive controls listed were spotted onto sample cards, eluted by the method of Klüber (unpublished Master’s Thesis) and quantified by multiplex-RT-PCR-ELISA. Multiplex-RT-PCR-ELISA conducted on the same controls not spotted onto cards serves as positive controls. All results were normalized in terms of pooled influenza A amplicons (20 µl/well) added to the microtiter plates

**Table 7:** Efficacy of multiplex-RT-PCR-ELISA conducted on positive controls eluted from sample cards

Pathogens^a^	*P. fal*	DENV	ZIKA	RVF	CHIKV	WNV	YFV
Elution Klüber method ELISA (OD_405_)	0.77 ± 0.1	0.59 ± 0.26	0.42 ± 0.25	1.49 ± 0.13	0.59 ± 0.24	0.38 ± 0.27	1.32 ± 0.54
Elution Klüber-Gruner method ELISA (OD_405_)	1.17 ± 0.25	0.41 ± 0.04	0.26 ± 0.07	1.44 ± 0.42	0.33 ± 0.07	0.33 ± 0.1	0.44 ± 0.23
Elution Gruner method ELISA (OD_405_)	0.6 ± 0.14	0.3 ± 0.02	0.25 ± 0.02	0.8 ± 0.1	0.24 ± 0.04	0.23 ± 0.04	0.47 ± 0.24
Not spotted ELISA (OD_405_)	2.31 ± 0.01	1.65 ± 0.07	2.12 ± 0.05	1.72 ± 0.09	1.27 ± 0.04	1.67 ± 0.11	2.04 ± 0.08

^a^Data, normalized based upon the OD_405_ values generated from pooled influenza A amplicons, are the means ± SEM obtained from triplicate plates shown in Fig. 3. OD readings ≥ 4-times the negative control (0.1 OD_405_) were judged to be positive

### Preservation capacity of Whatman^®^ 903 protein sample cards

The capacity of Whatman^®^ 903 protein sample cards to preserve samples obtained from 132 children admitted to the paediatric ward at BMC with a suspected *Plasmodium* infection depended upon the nature of the sample. DSS exhibited a 59.3% (95% CI [0.388–0.776]) preservation rate detected by multiplex-RT-PCR-ELISA performed on the eluate extracted from cards and 97.1% (95% CI [0.919–0.994]) specificity (Table 8). The preservation rate for DBS, on the other hand, was 100% (95% CI [0.872–1]) and the specificity was 98.1% (95% CI [0.933–0.998]). The consistency among preservation methods was excellent for whole blood spotted on sample cards (Cohen’s kappa statistic), but only good for serum samples on cards [32].

**Table 8:** Concordance rates of *Plasmodium* sp. spotted on cards

Sample card	Concordant^a^		Discordant^a^		% Concordance^b^		Cohen’s κ^c^
	+/+	−/−	±	−/ +			
Sera, n = 132	16/27	102/105	3/105	11/27	89.39%	0.634	
whole blood, n = 132	27/27	103/105	2/105	0/27	98.48%	0.955	

Sera and whole blood samples obtained from 132 children suspected of having malaria were spotted onto cards. Subsequently, the spots were eluted by the method of Klüber (unpublished Master’s Thesis) and quantified by multiplex-RT-PCR-ELISA

^a^Key: +/+, true positive; −/−, true negative; ± , false positive; −/+ , false negative

^b^Compared to the results of multiplex-RT-PCR-ELISA performed directly on the same, non-spotted patient sample

^c^Interrater reliability where κ 0.61–0.80 = substantial agreement and κ 0.81–0.99 = near perfect agreement

### Preservation and analysis of clinical samples

Blood samples, obtained from the 132 children referenced in the preceding paragraph, were spotted onto sample cards and dried for at least 24 h before storage under dry conditions; the duration of storage was 186 to 312 days (267 days median). Sera samples, mRDT test results, DBS and DSS samples were obtained from all 132 included patients. 122 blood smear results of the included patients could be obtained at BMC. In 10/132 cases blood smear evaluation was not performed or test results were not available.

Twenty-seven out of 132 patients’ sera were tested positive for *Plasmodium* sp*.* by multiplex-RT-PCR-ELISA. Following the above described extraction method, consequently 27/132 DBS and 19/132 DSS samples were found positive for *Plasmodium* sp*.* by multiplex-RT-PCR-ELISA. Each positive sample was confirmed by singleplex-PCR. 13.9% (17/122) of the blood smear tests had a positive finding for malaria parasites. The RDT from NADAL^®^ found twenty-eight out of 132 blood samples positive for *Plasmodium* sp*.* antigens.

The results of standard diagnostic test obtained at BMC were compared to those obtained by multiplex-valent-RT-PCR-ELISA. Microscopic examination of blood smears conducted at BMC lacked the sensitivity and specificity required to detect acute malaria infections (Table 9). The sensitivity and specificity of the mRDT NADAL^®^ and the multiplex-RT-PCR-ELISA, on the other hand, were approximately equal. Notably, the cause of high fever and malaria-like symptoms in the remaining paediatric patients found free of *Plasmodium* sp. was indeterminate; analysis of sera and DBS samples by multiplex-RT-PCR-ELISA failed to identify an aetiologic agent.

**Table 9:** Efficacy of standard diagnostic tools for detecting* Plasmodium* sp.

	Sensitivity (+/+)	Specificity (−/−)
Blood smear microscopy, n = 122	40.0% (10/25)	92.8% (90/97)
mRDT (NADAL^®^), n = 132	96.3% (26/27)	98.1% (103/105)

The presence of *Plasmodium* sp. in blood samples obtained from 132 children suspected of having malaria was assessed by blood smear microscopy and mRDT. The results were compared to those obtained for the same patient cohort that tested positive by multiplex-RT-PCR-ELISA, i.e., 27 of 132

## Discussion

The Department of Pediatric Immunology and Infectious Diseases at the University Mainz developed a multiplex-RT-PCR-ELISA for the comprehensive, laboratory-based surveillance of pathogens that cause acute respiratory infections [28]. A previously reported multiplex-RT-PCR panel combined with a microwell hybridization assay (multiplex-RT-PCR-ELISA) allows the simultaneous detection of 19 respiratory pathogens [14]. The feasibility of the multiplex-RT-PCR-ELISA was tested on clinical specimens and validated for diagnosis of respiratory tract infections; tests on culture supernatants were comparable to gold standard references. Descriptive epidemiological studies based upon results obtained by this method have been reported [33]. More than 30,000 samples have been analysed since 1996.

Here, it was demonstrated that positive controls for nine vector-borne pathogens can be detected simultaneously by a multiplex-RT-PCR-ELISA panel. Multiplex-RT-PCR-ELISA was tested with several replicates and found reproducible. 16 samples can be tested within around 10 h working time. This poses a major limitation implementing the multiplex-RT-PCR-ELISA into clinical routine of low-resource settings. The sensitivity limit of 1000 copies/ml is comparable with established multiplex-RT-PCR-ELISA approaches looking for Zika, Dengue and Chikungunya viral infections in clinical samples in Nicaragua [34]. Serologic markers are a powerful alternative for epidemiologic research of vector-borne diseases but are of limited value in diagnosing arbovirus coinfections. Extensive antigenic cross-reactivity between arboviruses contributes to a lack of specificity [35–37]. Moreover, *Plasmodium* sp. triggered polyclonal B-lymphocyte activation during cases of malaria can lead to ambiguous serologic results [38–40].

Tanzania must be added to the list of countries where *Plasmodium* sp. and arboviruses are endemic, and coinfections are likely to occur [41, 42]. Diagnostic tools like multiplex-RT-PCR-ELISA can differentiate the pathogens that cause acute febrile disease and should be considered as an approach in countries where a wide variety of mosquito-borne diseases are prevalent. Multiplex-RT-PCR-ELISA panels are easily expanded and modified, and thereby adapted to current epidemiologic challenges. Arbovirus infections usually have a short viremic phase, lasting 5–7-days and preceding clinical symptoms. As such, there is a risk of collecting blood samples too late, causing false negative results and an underestimated prevalence of these pathogens. To overcome this limitation, specific serologic tests capable of distinguishing precisely between different flaviviruses might be a reasonable addition to determining the prevalence of arboviruses in SSA-regions [43]. Still, alphaviruses like CHIKV and ONNV remain a diagnostic challenge in terms of detection with serological methods.

Microscopic examination of blood smears is the standard diagnostic approach for suspected cases of malaria at tertiary health facilities, such as BMC. Only 21% of the presumptive malaria cases in the presented cohort could be confirmed by multiplex-RT-PCR-ELISA or the RDT. Incongruent malaria diagnostics reveal a significant limitation in daily clinical routine. RDT NADAL^®^ exhibited 96% sensitivity in detecting acute malaria infections and was very specific (98%). In contrast, examination of blood smear performed at BMC detected only 4 out of 10 cases of malaria. These findings underline how diagnostic approaches with low sensitivity performance lead to misassumption of malaria cases. Studies conducted in Northern Tanzania concluded that using microscopy testing malaria is over-diagnosed and malaria-like febrile illnesses caused by other mosquito-borne pathogens such as CHIKV are underestimated [44]. Parallelly, microscopy of blood films misses clinically relevant cases of low-density parasitaemia [45–47]. The RDT has proven to be highly accurate and cost-effective for the Tanzanian government [3]. Significant discrepancies in the results of diagnostic tests for malaria (i.e., blood smear microscopy and RDT *versus* genus-specific PCR) were documented in Dar es Salaam, Tanzania [48]. Remarkably, NADAL^®^ RDT used in the study reported herein showed greater sensitivity than did Paracheck-Pf^®^ RDT compared to RT-PCR as the gold standard in similar studies [49]. The data reported herein should provide a rational approach for the targeted use of anti-malarials and antibiotics, thus reducing the potential overuse of both medications. Notably, the WHO goal of “keeping artemisinin-based drugs an effective treatment against malaria infections” (2011) correlates with this objective [50].

A wide range of vector-borne pathogens spotted on sample cards, including *Plasmodium* sp*.*, DENV, YFV, RVF and CHIKV, was detected by multiplex-RT-PCR-ELISA. A variety of factors, e.g., storage conditions such as temperature and humidity, can influence the capacity of these cards to preserve vector-borne pathogens. Extraction with nuclease-free water optimized recovery of material in DBS from sample card in the study reported herein. This finding correlates with results of other investigators who reported the highest yield of DENV-positive clinical specimens spotted on sample cards was obtained by nuclease-free water extraction [51]. Indeed, nuclease-free water is already used for extracting DENV antibodies from DSS [52]. However, while extraction at a high temperature (72 °C) favoured recovery of nucleic acids from samples spotted on cards in our study, such high temperatures would limit recovery of antibodies for subsequent diagnostic tests. Moreover, ZIKV and WNV spotted onto sample cards in the study reported here were not readily extracted in nuclease-free water at 72 °C and quantified, indicating the obligate need to conduct additional studies to maximize the recovery of a diverse pathogen population and its components spotted on sample cards.

In 2012, the WHO declared a global strategy to reduce the burden of vector-borne pathogens like DENV by 2020 [53]. A systematic literature review of DENV infections in Tanzania concluded that DENV is endemic and must be regarded as a relevant health issue [8]. Herein, it was documented that Whatman^®^ 903 protein sample cards represent a feasible approach to collecting presumptive, vector-borne disease samples, especially in rural areas. The degree to which specimens were preserved on sample cards differed significantly dependent upon the nature of the sample. Only ~ 60% of *Plasmodium*-positive samples were preserved and recovered from DSS; all positive samples preserved as DBS, however, could be detected by multiplex-RT-PCR-ELISA. Similarly, Prado et al. reported the same discordant preservation of DENV-positive specimens eluted from cards and quantified by PCR, i.e., 100% specificity for DBS and only 47% for DSS [51].

Grüner et al*.* recommended storing DBS samples at room temperature for up to 2 weeks [17]. Remarkably, the data obtained in the present study indicates that the capacity to store *Plasmodium* sp. on sample cards could exceed 10 months, confirming the report of other investigators concerning the extended storage capacity of sample cards [54]. The ability to preserve viral components such as RNA at room temperature under dry conditions for a long period of time makes DBS on sample cards an extremely suitable method for preserving diagnostic specimens under challenging conditions. Establishment of DBS-based, multiplex-PCR-ELISA in referring SSA centres would simplify storage conditions and enable transport of samples to laboratories which are properly equipped for testing. An additional advantage of DBS is that only small quantities of blood are needed, a tremendous benefit when testing children.

Models, which consider the influence of climate on the distribution of vector habitats in the future, warn that outbreaks of arbovirus infections will increase in Tanzania within the next few years [55]. The Lake Victoria Region was identified as an area at risk [56]. Systematic surveillance would enable the early detection of arbovirus outbreaks and simplify therapeutic approaches, e.g., antipyretics, fluids or transfusion of blood components. Consequently, DBS and blood sample testing should continue in the Lake Victoria Region. Future aspects of epidemiologic research should also consider improved methods to extract and analyse serologic markers from sample cards [52, 57].

## Conclusions

Point of care diagnostic tests comprise a promising area of research that has proven to reduce the prescription of non-indicated antibiotics and anti-*Plasmodium* drugs to children with acute, febrile diseases in Tanzania that are unrelated to malaria [58]. Indeed, smart ELISA platforms, which are capable of detecting serologic markers of DENV-infections, exhibit up to 95% sensitivity in clinical settings [59]. Unfortunately, the multiplex-RT-PCR-ELISA developed for the present study failed to determine the cause of malaria-like symptoms in our paediatric patient cohort found free of *Plasmodium* sp. The cyclic nature of arboviral prevalence, fluctuating between outbreaks and low endemicity, and the time gap between viremia and clinical manifestation could be factors contributing to the absence of arboviruses in the data presented. Additionally, other common causes of acute fever such as viral respiratory tract infections, gastrointestinal infections, urinary tract infections and typhoid fever were not surveyed. As a consequence, the long-term goal of the present study is to continue monitoring and identifying the distribution of mosquito-transmittable pathogens most prevalent in SSA by multiplex-RT-PCR-ELISA. Once this is achieved, improved PCR-techniques such as loop mediated isothermal amplification (LAMP) will pave the way to well-targeted, robust and easy-to-use testing tools [60].

## Data Availability

The datasets used and/or analysed during the current study are available from the corresponding author on reasonable request.

## Abbreviations

ARI: Acute respiratory infection
BMC: Bugando Medical Centre
CHIKV: Chikungunya virus
CI: Confidence interval
CUHAS: Catholic University of Health and Allied Sciences
DBS: Dried blood spots
DSS: Dried serum spot
DENV: Dengue virus
ELISA: Enzyme-linked immunosorbent assay
HBV: Hepatitis B virus
HCV: Hepatitis C virus
HIV: Human immunodeficiency virus
RDT: Malaria rapid diagnostic test
RT-PCR: Reverse transcriptase-polymerase chain reaction
NIMR: National Institute of Medical Research
ONNV: O’nyong nyong virus
RVFV: Rift Valley fever virus
SFV: Semliki forest virus
SSA: Sub-Saharan Africa
WHO: World Health Organization
WNV: West Nile virus
YFV: Yellow fever virus
ZIKV: Zika virus
